# Supplementary material for: Allosteric ligand–aptamer complexes orchestrate supramolecular or transient catalytic, transcription and fibrinogenesis processes
Source: Chem Sci. 2026 Feb 2;17(12):6086–98. doi: 10.1039/d5sc09098a (PMC12863195; doi:10.1039/d5sc09098a)
Supplement: SC-017-D5SC09098A-s001 [file SC-017-D5SC09098A-s001.pdf]

Supplementary Information (SI) for Chemical Science.
This journal is © The Royal Society of Chemistry 2026

Supporting information for

Allosteric Ligand-Aptamer Complexes Orchestrate Supramolecular or Transient Catalytic, Transcription and Fibrinogenesis Processes

Diva Froim[a], Hadar Amartely[b], Jiantong Dong[a], Eli Pikarsky[c], Itamar Willner[a*]*

[a] Institute of Chemistry, The Hebrew University of Jerusalem, Jerusalem 91904, Israel.

[b] Wolfson Centre for Applied Structural Biology, The Hebrew University of Jerusalem, Jerusalem 91904, Israel

[c] The Lautenberg Center for Immunology and Cancer Research, IMRIC, The Hebrew University of Jerusalem, Jerusalem 91120, Israel

*Email: jtdong2025@nankai.edu.cn; itamar.willner@mail.huji.ac.il

Experimental Section

Materials

DNA oligonucleotides, substrate/quencher-modified substrates and RNA were purchased through Integrated DNA Technologies Inc. T7 RNA polymerase (50,000 units/mL, 0.8 μM), ribonucleotide (NTP) Mix (GTP, ATP, UTP and CTP, each 25 mM), separated ribonucleotides (UTP, GTP, CTP, each 100mM) and 10 × RNAPol reaction buffer (400 mM Tris-HCl, 60 mM $MgCl_2$, 10mM DTT, 20 mM spermidine, pH 7.9 @ 25 °C) were purchased from New England BioLabs Inc. Thrombin from human plasma (≥ 2,000 NIH units/mg protein, MW = 37.4 kDa), fibrinogen from human plasma (50–70% protein), adenosine deaminase (1,000 units/mL), melamine, adenosine, $MgCl_2$, tris acetate, BSA, DTT, RNAse free water and Malachite Green (MG) were purchased from Sigma-Aldrich.

Instrumentation

DNA concentrations were determined by UV-1900 spectrophotometer (Shimadzu). Time-dependent fluorescence changes were recorded with a Cary Eclipse Fluorometer (Agilent Technologies) using a quartz cuvette with 10-mm path length supplied by Hellma Analytics, FAM was excited at 496 nm and emission was recorded at 520 nm. Malachite green was excited at 632 nm and emission was recorded at 650 nm. Light scattering changes were recorded at 650 nm using a plastic cuvette with 10-mm path length purchased from Brand GMBH, Wehrheim, Germany. Isothermal titration calorimetry measurements were performed on the PEAQ-ITC instrument (Malvern).

Melamine-mediated stabilization of Mg^{2+}-dependent DNAzyme subunits

DNAzyme reaction mixtures consisted of 1 μM of each DNAzyme subunits strands D_m and E_m, 20 mM Tris acetate buffer pH 7.9, 40 mM $MgCl_2$ and 2.25 μM BSA with varying concentrations of melamine. The mixture was incubated at room temperature for 1 hour. After the addition of 1 μM of the substrate strand, S, time-dependent fluorescence changes generated by the cleavage of the modified fluorophore/quencher substrate were monitored at 25°C (600V). The temporal concentrations of the free fluorophore were quantified using the calibration curve shown in Figure S1.

Allosteric melamine-mediated inhibition of thrombin-induced fibrinogenesis

Reaction mixtures consisted of 1 μM of each of the DNA subunits in 40 mM Tris acetate buffer pH 7.9 and 40 mM $MgCl_2$ with varying concentrations of melamine. Subsequent to a 1-hour incubation at room

temperature, 5 nM thrombin was added to the solution and incubated at room temperature for 15 minutes. To evaluate thrombin coagulation activity, 10 mg/ml fibrinogen was added and the intensity changes of scattered light caused by the formation of the fibrin network were immediately monitored at 25°C (500V).[1]

Transcription of the Malachite Green RNA aptamer by the allosteric melamine-triggered transcription machinery

For the melamine transcription experiments 20 µM of the template strands N_m and T_m were annealed in 1×RNAPol reaction buffer at 85°C for 5 minutes then cooled down to 25°C over 15 minutes.

A reaction mixture with varying concentrations of melamine, 0.2 µM annealed N_m/T_m, 0.2 µM P_m strand, 1×RNAPol reaction buffer, 20 mM $MgCl_2$, 5 mM DTT, 0.5 mM NTPs and 2 µM MG was incubated at room temperature for 1 hour. The system was activated by the insertion of 1.5 $U \cdot \mu l^{-1}$ T7 RNAP, time-dependent fluorescence changes generated by the binding of MG/MG RNA aptamer were measured at 35°C (900V). The temporal concentrations of the malachite green RNA aptamer were quantified using the calibration curve shown in Figure S5.

Allosteric adenosine/ADA-modulated, transient, dissipative, inhibition of thrombin-induced fibrinogenesis

The adenosine induced inhibition of fibrinogenesis reaction mixtures contained 1 µM of each of the DNA subunits in 40 mM Tris acetate buffer pH 7.9 and 40 mM $MgCl_2$ was incubated for 1 hour at room temperature with variable concentrations of adenosine. Subsequent the incubation, 5 nM thrombin was added to the solution and incubated at room temperature for 15 minutes. To evaluate thrombin coagulation activity, 10 mg/ml fibrinogen was added and the intensity changes of scattered light caused by formation of the fibrin network were immediately monitored at 25°C (500V).[1]

To probe the temporal activity of thrombin within the dissipative system containing ADA, a reaction mixture was prepared, one sample was withdrawn prior to the addition of adenosine. Next, adenosine was added and the reaction mixture was allowed to incubate for 1 hour at room temperature. Following incubation, variable concentrations of ADA were added, aliquots of 90µl were withdrawn from the reaction mixture at defined time intervals and incubated with 5 nM thrombin for 15 minutes at room temperature. To evaluate thrombin coagulation activity, 10 mg/ml fibrinogen was added and the light-

scattering changes caused by formation of the fibrin network were immediately monitored at 25°C (500V).[1]

Transient, dissipative RNA transcription by the allosteric adenosine-activated, ADA-modulated, transcription machinery

For the adenosine-activated transcription experiments 20 μM of the template strands N_a and T_a were annealed in 1×RNAPol reaction buffer at 85°C for 5 minutes then cooled down to 25°C over 15 minutes. The fluorophore and quencher modified DNA strands were annealed following the same procedure.

Reaction mixtures with varying concentrations of adenosine, 0.2 μM annealed N_a/T_a strands, 0.2 μM P_a strand, 2 μM annealed F/Q strands, 1×RNAPol reaction buffer, 20 mM $MgCl_2$, 5 mM DTT and 3 mM NTPs were incubated at room temperature for 1 hour. The system was activated by the insertion of 1.2 $U \cdot \mu l^{-1}$ T7 RNAP, time-dependent fluorescence changes generated by the displacement of the quencher strand by the RNA product were measured at 33°C (600V).

Dissipative transcription experiments with ADA were prepared and measured in the same manner. Following activation by T7 RNAP, varying concentrations of ADA were added to the system incubated with 2 mM adenosine or alternatively, 0.025 $U \cdot ml^{-1}$ ADA was added to the system incubated with different concentrations of adenosine. The temporal concentrations of the RNA generated by transcription machinery were quantified using the appropriate calibration curve shown in Figure S7.

Isothermal Titration Calorimetry (ITC) experiments

ITC reaction solutions were prepared in 20 mM Tris acetate pH 7.9 and 40 mM $MgCl_2$ (Total volume 300μl). Each experiment consisted of 17 injections of 2.3μl melamine into DNA aptamer solutions ranging from 13 to 15 μM for the intact and split melamine aptamer with added subdomain a/a', and 37 μM for the split aptamer lacking the added subdomain, stirred at 750 rpm and maintained at 25°C. A delay of 150 s between injections was allowed for equilibration. All experiments were corrected for the heat of dilution of the titrant. Data was fit to a one set of binding sites binding model using MicroCal PEAQ-ITC Analysis Software. Measurement was performed using a MicroCal PEAQ-ITC instrument (Malvern).

ITC experiments characterizing the adenosine-induced stabilization of the anti-thrombin aptamer subunits were conducted similarly to the melamine/aptamer experiments, except DNA aptamer

solutions ranged from 30 μM to 40 μM and were prepared in buffer solutions consisting of 40 mM Tris acetate pH 7.9 and 40 mM $MgCl_2$. The delay between injections was extended to 350 s.

Table S1. Nucleic acid strands used in this study

Name	Sequence (5' – 3')
E_m	5′-ACCTTTAGGGGGTGTGC**CACCCATGT**TCCTGA-3′
D_m	5′-CTGTTC**AGCGAT**GCACACCGATGGCGGTCCTGTAGGT-3′
S	5’-FAM-TCAGGATrAGGAACAG-BHQ1-3’
G_m	5′-AGGGTGGTGGCGAAAGCACACCGATGGCGGTCCTGTAGGT-3′
H_m	5′-ACCTTTAGGGGGTGTGCAAACGCCTAGGTTGGGT-3′
N_m	5’-TAATACGACTCACTATAGGGATCCCGACTGGCGAGAGCCAGGTAACGAATGGATCC-3’
T_m	5’-GGATCCATTCGTTACCTGGCTCTCGCCAGTCGGGATCCCTATAGTGAGTCTTAGGGGGTGTGC-3'
P_m	5’-GCACACCGATGGCGGTCCTGTGTATTA-3'
R_1	5′- GGAUCCCGACUGGCGAGAGCCAGGUAACGAAUGGAUCC -3′
G_a	5′-AGGGTGGTGGCGAACTGCGGAGGAAGGT-3′
H_a	5′-ACCTGGGGGAGTATGATCGCCTAGGTTGGGT-3′
N_a	5’-ACCTGGGGGAGTATTAATACGACTCACTATAGGGTTCTCCTCTGCGTTGTTGTTTG-3’
T_a	5’-CAAACAACAACGCAGAGGAGAACCCTATAGTGAGTCG-3'
P_a	5’-TATTATGCGGAGGAAGGT-3’
R_2	5′- GTTCTCCTCTGCGTTGTTGTTTG -3′
F	5′-FAM-CAAACAACAACGCAGAGGAGAAC-3′
Q	5′-GTTCTCCTCTGCGTT-BHQ1-3′
Note	DNAzyme subunits are marked in bold. The ribonucleotide modification is colored in red. The T7 promoter is colored in blue and thrombin aptamer subunits are colored in orange.

Table S2. Nucleic acid strands used in Isothermal Titration Calorimetry experiments

Name	Sequence (5' – 3')
Intact Mel aptamer	5'-GCACACCGATGGCGGTCCTGTTTAGGGGGTGTGC-3'
Split Mel aptamer – 0nt	(a) 5'-GCACACCGATGGCGGTCCTGT-3'<br>(b) 5'-TTAGGGGGTGTGC-3'
Split Mel aptamer – 4nt	(a) 5'-GCACACCGATGGCGGTCCTGTAGGT-3'<br>(b) 5'-ACCTTTAGGGGGTGTGC-3'
Split Ade aptamer – 0nt	(a) 5'-AGGGTGGTGGCGTGCGGAGGAAGGT-3'<br>(b) 5'-ACCTGGGGGAGTATCGCCTAGGTTGGGT-3'
Split Ade aptamer – 3nt	(a) 5'-AGGGTGGTGGCGAACTGCGGAGGAAGGT-3'<br>(b) 5'-ACCTGGGGGAGTATGATCGCCTAGGTTGGGT-3'
Note	Added bases are underlined.

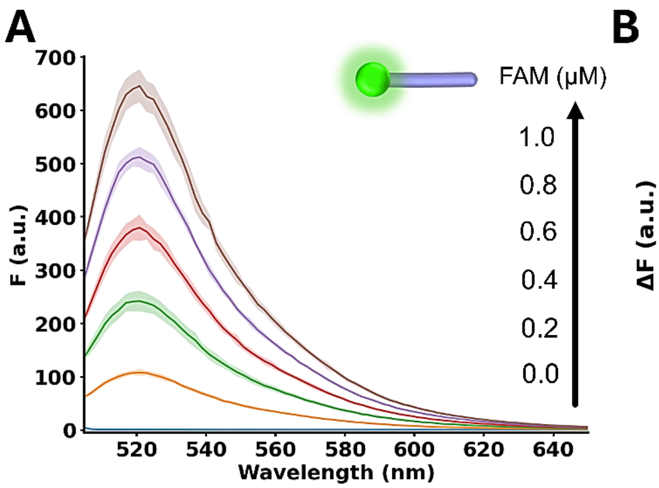

Figure S1. (A) Fluorescence spectra of different fluorophore (FAM) concentrations (λ_{ex}= 496 nm). (B) Derived calibration curve corresponding to the fluorescence intensities increasing as a function of the fluorophore concentration at λ_{em}= 520 nm. R^2=0.999.

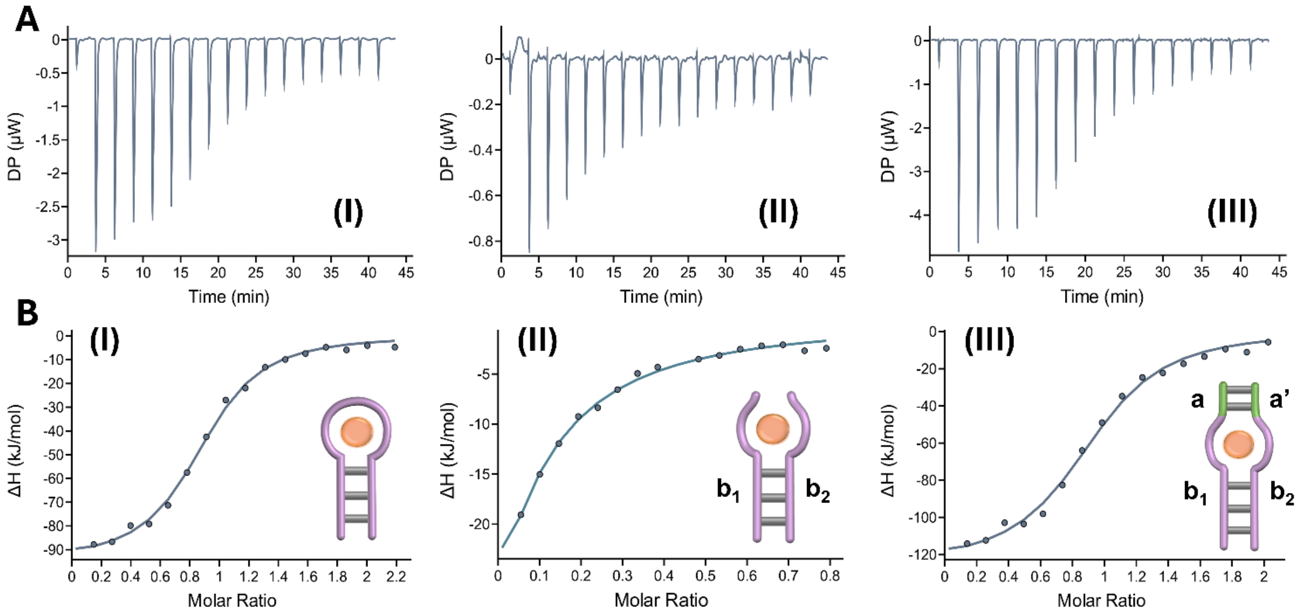

Figure S2. Isothermal titration calorimetry (ITC) experiments determining the K_d, stoichiometry and thermodynamics of the binding of melamine to the intact melamine aptamer and the split melamine aptamer with and without added base pairing. (A) Thermograms of melamine titrations into an intact melamine aptamer (panel I), split melamine aptamer subunits (panel II) and split melamine subunits with the added of subdomain a/a' (panel III). (B) Integrated curves representing the total heat exchanged derived from (A), intact aptamer (panel I), split aptamer subunits (panel II) and split aptamer subunits with subdomain a/a' (panel III).

Table S3. Isothermal titration calorimetry (ITC) analysis of melmaine/aptamer

**DNA**	**K_d** (μM)	**N** (sites)	**ΔG** (kJ·mol^{-1})	**ΔH** (kJ·mol^{-1})	**-TΔS** (kJ·mol^{-1})
Intact aptamer	0.800 ±0.139	0.869 ±0.02	-34.8	-95.1 ±2.89	60.3
Split aptamer – 0bp	11.1 ±2.09	0.022 ±0.048	-28.3	-335 ±751	307
Split aptamer – 4bp	0.890 ±0.123	0.902 ±0.017	-34.7	-125 ±3.15	90.3

Design of the melamine-induced allosteric operation of the Mg^{2+}-ion dependent DNAzyme

To successfully accomplish the melamine-induced allosteric stabilization of aptamer subunits D_m/E_m of the Mg^{2+}-ion-dependent DNAzyme, it is essential to adapt the following requirements.

- The D_m/E_m aptamer subunits should form a stable melamine/aptamer subunits complex.
- Background DNAzyme activity stemming from independent formation of the supramolecular D_m/E_m complex, in the absence of melamine should be eliminated.
- The ΔG^0 values should be estimated and the structures should be optimized to follow the requirements.
- The ΔG guided design of the sequences should then be experimentally validated to confirm optimal melamine-induced allosteric operation of the DNAzyme.

Isothermal Titration Calorimetry (ITC) is an effective method to estimate the ΔG values of melamine/aptamer subunits complexes.

a) The intact melamine/aptamer complex exhibits a $\Delta G = -34.8\ kJ\cdot mol^{-1}$ (Fig. S2, panel I)
b) The cleaved aptamer subunits D_m/b_1 and E_m/b_2 in the presence of melamine yield a complex with substantially lower stability, $\Delta G = -28.3\ kJ\cdot mol^{-1}$ (Fig. S2, panel II), suggesting that the allosteric stabilization of the DNAzyme will be inefficient.
c) Extension of the aptamer subunits D_m/b_1 and E_m/b_2 with added base pair tethers could synergistically stabilize the melamine/aptamer subunits. We find that extension of the aptamer subunits by four complementary base pairs, a/a', lead in the presence of melamine to a supramolecular melamine-$D_m/b_1/a+E_m/a'/b_2$ complex exhibiting a $\Delta G = -34.7\ kJ\cdot mol^{-1}$ (Fig. S2, panel III), comparable to the intact melamine/aptamer complex shown in Panel I.

Accordingly, the allosterically engineered aptamer subunits stabilizing the Mg^{2+}-dependent DNAzyme units were selected to include the four complementary base extension a/a'.

Indeed, the experimental results shown in Figure 1(C) demonstrate a zero-background DNAzyme activity in the absence of melamine.

Note, that extension of D_m/b_1 and E_m/b_2 with longer complementary base pair a/a' sequences yield a significantly stable supramolecular complex, even in the absence of melamine, supporting background DNAzyme activity. For example, a 5-base complementary extension of a/a' tethers reveals a significant background DNAzyme activity.

It should be further addressed that NUPACK's[2] (version dna04.1) predicted free energy values of potential duplexes between the 4 or 5 base complementary extension of a/a', in the absence of melamine, do not support the formation of stable duplexes ($\Delta G \geq -9$ kcal/mol), albeit the 5-base structure showed in the absence of melamine a significant background catalytic activity. Thus, the design of the optimized sequences relied on ITC measurements and complementary experimental validations.

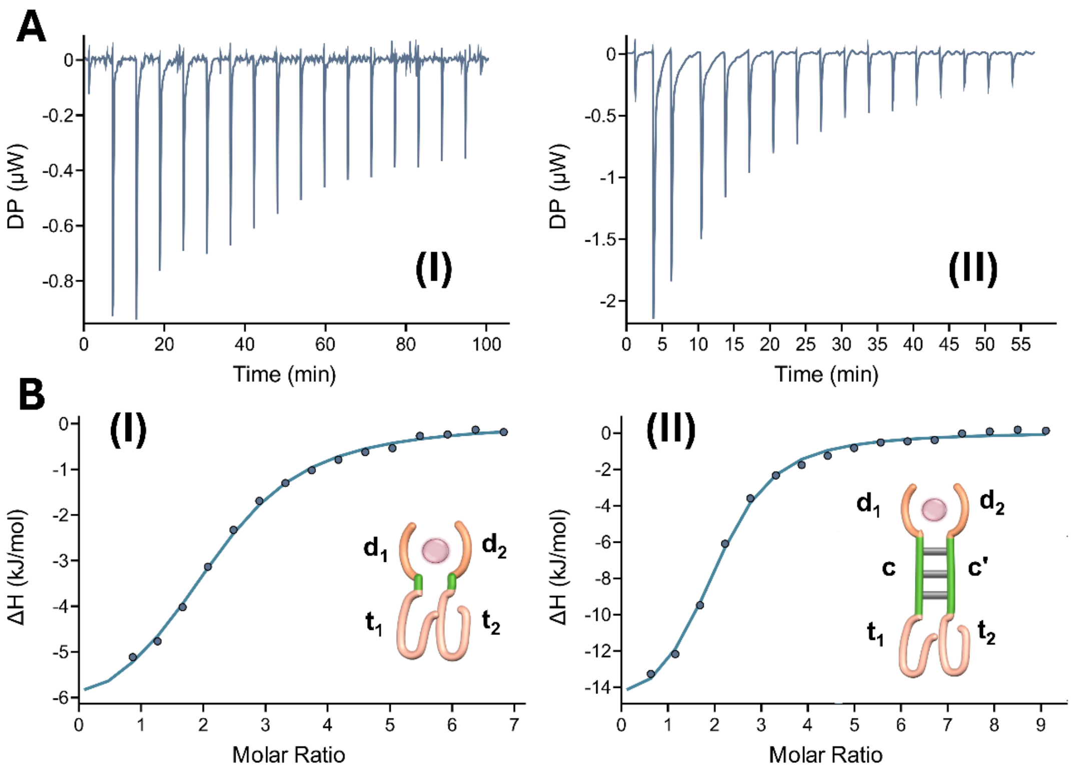

Figure S3. Isothermal titration calorimetry (ITC) experiments characterizing the binding of adenosine to the aptamer subunits, d_1 and d_2 with the added subdomain c/c' and without. (A) Thermograms of adenosine titrations into the $d_1/t_1+d_2/t_2$ construct (panel I) and $d_1/c/t_1+d_2/c'/t_2$ construct (panel II). (B) Integrated curves representing the total heat exchanged derived from (A), without the c/c' subdomain (panel I) and with the added c/c' (panel II).

Design of the adenosine/aptamer subunits circuit for allosteric inhibition of thrombin

As before, the design principle of the adenosine/aptamer subunits circuit for the allosteric inhibition of thrombin is based on Isothermal Titration Calorimetry (ITC) measurements and coupled experiments validating the ITC predictions.

1. In the first step the binding capacities of the adenosine/aptamer subunits $d_1/t_1+d_2/t_2$, Figure S3 were evaluated by ITC measurements, Fig. S3(A) and Fig. S3(B), panel I. Weak adenosine-induced formation of the adenosine/aptamer subunits was observed K_d = 14.1 ±1.3 μM. Experiments probing the inhibition of thrombin by this construct confirmed that these aptamer subunits did not lead to sufficient allosteric inhibition of thrombin.
2. Accordingly we probed the possibility to enhance the stability of the apatmer subunits via the introduction of additional base pairing stabilizing the structure – $d_1/c/t_1+d_2/c'/t_2$. ITC measurements, Fig. S3(A) and Fig. S3(B), panel II, confirmed that the resulting supramolecular adenosine/aptamer subunits structure was indeed stabilized by the two subunits, K_d = 7.28 ±0.82 μM. Moreover, in the absence of adenosine, no binding between the aptamer subunits proceeded.
   Indeed, experiments probing the inhibition of thrombin by the $d_1/c/t_1+d_2/c'/t_2$ strands in the presence of adenosine showed effective inhibition of thrombin, whereas in the absence of adenosine, no inhibition was observed, indicating the allosteric function of adenosine guiding the inhibition of thrombin.
3. Further experiments extending the bridging domain c/c' of the two subunits with three base pairs, demonstrated allosteric adenosine-driven inhibition of thrombin. Nevertheless, control experiments in the absence of adenosine demonstrated background inhibition of thrombin.
   Thus, the design principle employing ITC experiments and complementary validating inhibition experiments provide useful tools to design optimized circuits for the allosteric inhibition of thrombin.

   It should be noted that NUPACK's (version dna04.1) free energy predictions regarding the stability of the potential duplexes, consisting of additional 2 or 3 base pairing in strands $d_1/c/t_1+d_2/c'/t_2$, **in the absence of adenosine**, did not predict distinguishable differences in the formation of stable duplexes (ΔG = -16.7 and -17.6 kcal/mol, respectively). The ITC measurements, in the presence of adenosine showed significant differences in the resulting K_d values that were experiemntally validated, thus enabling the optimization of the selected strands. Thus, coupling ITC and experimental validation to predict the stability of allosteric complexes is a robust design principle.

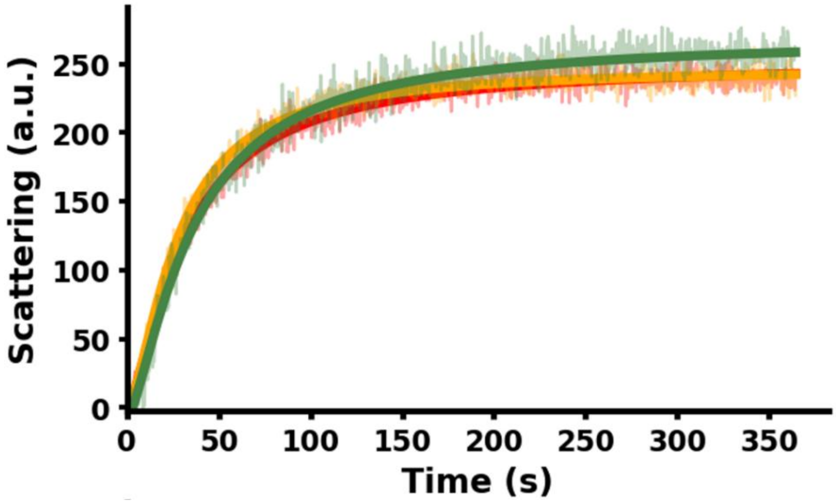

Figure S4. Temporal light scattering probing thrombin activity in the absence of DNA without additives and with 2 mM of adenosine or melamine. Samples were analyzed following the procedure described previously. Data are means±SD, N=3.

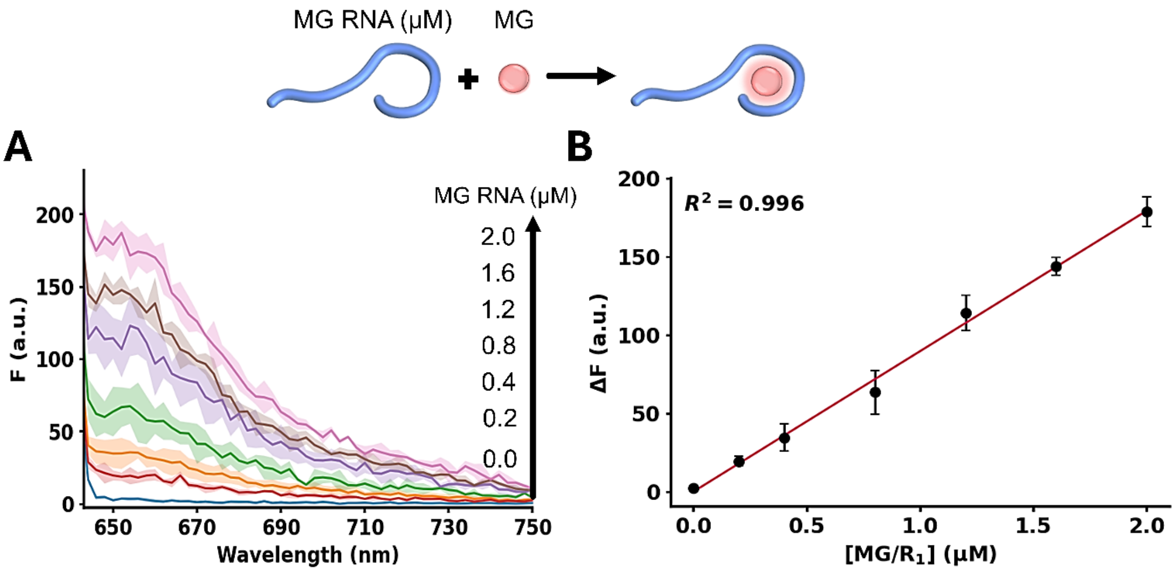

Figure S5. (A) Fluorescence spectra obtained from the Malachite Green (MG)/RNA aptamer complex, in the presence of variable concentrations of the RNA aptamer (λ_{ex}= 632 nm). (B) Derived calibration curve corresponding to the fluorescence intensities of the MG/RNA aptamer complex as the concentration of the malachite green RNA aptamer increases at λ_{em}= 650 nm. R^2=0.996.

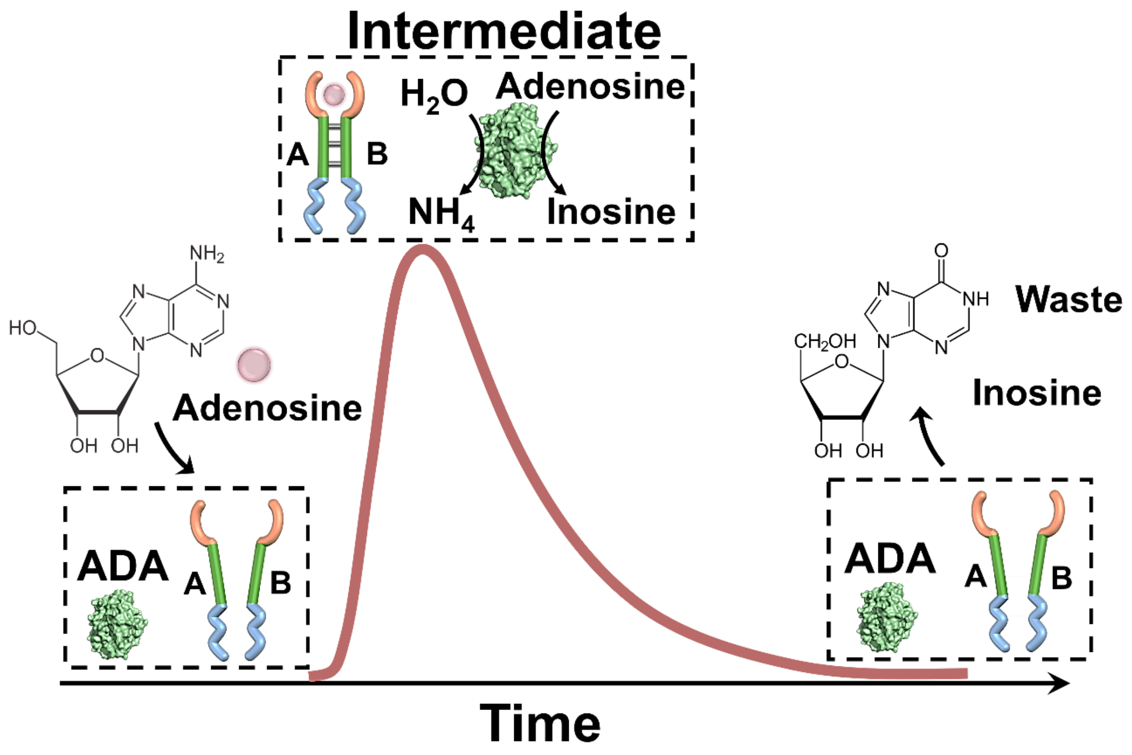

*** The concept is applicable to other dissipative ligand/aptamer complexes coupled to ligand degrading enzymes such as uric acid/uricase and acetylcholine/acetylcholinesterase.**

Figure S6. General scheme of the dissipative transient operation of a DNA-based reaction circuit including the split adenosine aptamer subunits present in strands A and B that are unable to form a stable duplex. Addition of adenosine activates the system, creating the intermediate product adenosine/A/B interstrand complex. However, adenosine deaminase (ADA) present in the reaction module, deaminates adenosine to inosine, which lacks affinity to the adenosine aptamer subunits, leading to the separation of A and B and the dissipative deactivation of the system.

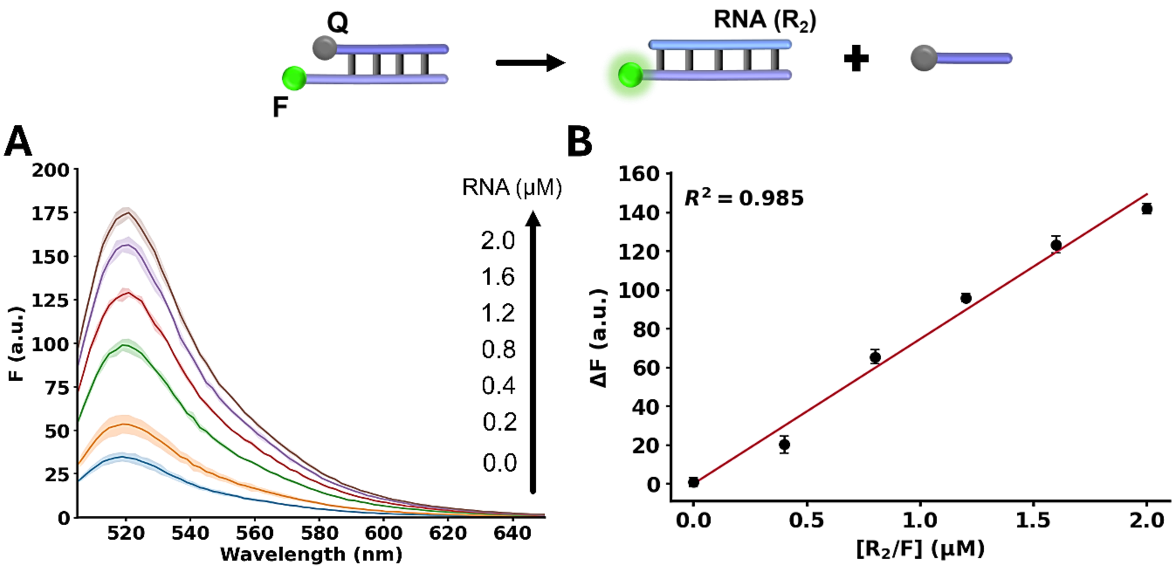

Figure S7. (A) Fluorescence spectra of FAM-labeled F DNA strand (2.0 μM) generated by the addition of different concentrations of R_2 resulting in the displacement of the BHQ1-modified Q DNA strand (λ_{ex}= 496 nm) from the F/Q duplex. (B) Derived calibration curve corresponding to the fluorescence intensities increasing as the concentration of R_2 increases at λ_{em}= 520 nm. R^2=0.985.

References

[1] J. Wang, Y. Wei, X. Hu, Y.-Y. Fang, X. Li, J. Liu, S. Wang and Q. Yuan, *J. Am. Chem. Soc.* 2015, **137**, 10576–10584.

[2] J. N. Zadeh, C. D. Steenberg, J. S. Bois, B. R. Wolfe, M. B. Pierce, A. R. Khan, R. M. Dirks, N. A. Pierce. NUPACK: analysis and design of nucleic acid systems. *J Comput Chem*, **32**:170–173, 2011.
